# UNPACKING MILD COGNITIVE IMPAIRMENT/YOUNG ONSET DEMENTIA IN THE WORKPLACE: EMPLOYER VERSUS EMPLOYEE

**DOI:** 10.1093/geroni/igac059.493

**Published:** 2022-12-20

**Authors:** Philip Taylor, AnneMarie Levy, Allison Gibson

## Abstract

The symposium includes four studies exploring the impact of mild cognitive impairment and/or young onset dementia on the workplace, and area of increasing interest due to the aging workforce and government interest in prolonging work life. The papers adopt a rare, multi-level approach by examining both employee and employer perspectives, and how the interaction of both individual and organizational factors impact their self-reported outcomes. Following the paper presentations, Dr. Allison Gibson will lead a paper discussion. The goal of the symposium is to foster a research conversation amongst the presenters and symposium participants interested in exploring overlaps, potential for multi-disciplinary collaboration and future research into resolution of workplace and workforce optimization with worker satisfaction that are identified with progressive cognitive impairment.

